# Characteristics and prognosis of patients with *Edwardsiella tarda* bacteremia at a single institution, Japan, 2005–2022

**DOI:** 10.1186/s12941-022-00548-w

**Published:** 2022-12-07

**Authors:** Kohei Hasegawa, Murata Kenya, Kumiko Suzuki, Yoshihiko Ogawa

**Affiliations:** 1 Department of Infectious Diseases, Sakai City Medical Center, Ebaraji 1-1-1, Sakai, Osaka Japan; 2 Department of Clinical Laboratory, Sakai City Medical Center, Sakai, Osaka Japan

**Keywords:** *Edwardsiella tarda*, Zoonosis, Bacteremia, Epidemiology, Hepatobiliary infection

## Abstract

**Background:**

*Edwardsiella tarda* is a member of Enterobacteriaceae isolated from freshwater and sea. *E. tarda* infection in humans commonly causes gastroenteritis, but rarely causes bacteremia. However, few studies have described the clinical features of *E. tarda* bacteremia (ETB); therefore, we conducted a case review in our hospital.

**Methods:**

We conducted a single-center, retrospective descriptive study using electronic medical records. Patient and microbial features were extracted and evaluated for 30- and 90-day mortality rates.

**Results:**

From April 2005 to April 2022, the total set of blood cultures positive for any microorganisms was 9368, 38 of which were positive for *E. tarda*. Underlying cancer was observed in 65.8% of patients. The most common source of bacteremia was cholangitis, followed by cholecystitis, and endoscopic or surgical drainage was performed in almost all cases. Diarrhea was observed in only one patient, and there were no cases in which gastroenteritis was the source of bacteremia. All cases, except for one, were susceptible to all β-lactams, such as ampicillin. The 30- and 90-day overall mortality rates were 8.6% (3/35) and 25.8% (8/31). Of these, 75% patients died because of cancer progression after successful ETB treatment.

**Conclusion:**

ETB may occur in patients with malignant underlying conditions. Biliary tract infections are common in ETB cases, whereas gastroenteritis may be an atypical cause of bacteremia. This study suggests that although the mortality rate for ETB at 30 day was low, it increased at 90 day as a result of the development of unfavorable underlying conditions.

**Supplementary Information:**

The online version contains supplementary material available at 10.1186/s12941-022-00548-w.

## Introduction

*Edwardsiella* spp. are a gram-negative, oxidase-negative, catalase-positive, facultative anaerobes belonging to Enterobacteriales. *Edwardsiella tarda* is commonly isolated from freshwater and marine organisms, including turtles, fish, pelicans, crocodiles, seals, frogs, snakes, and lizards. *E. tarda* causes emphysematous rot in catfish and chronic enteritis in penguins [1]. Humans appear to be accidental hosts of *E. tarda*, and gastrointestinal diseases are the most common source, accounting for 83% of the cases [1]; extraintestinal infections, such as bacteremia, cholangitis, infective aneurysms, gynecologic infections, and skin and soft tissue infections, have also been reported [1–4]. The 30-day mortality rate for *E. tarda* bacteremia (ETB) was reported to be 11.5% in a case series [5], and the 90-day mortality rate was reported to be 44.6% in a literature review [6]. However, studies focusing on epidemiological information, treatment options, and prognosis of ETB have been scarce. Therefore, we conducted a retrospective descriptive review of patients with ETB in our institution to clarify the clinical characteristics of ETB.

## Methods

### Settings

This retrospective descriptive review of patients with ETB was conducted at Sakai City Medical Hospital, in Osaka, Japan, a 487-bed tertiary care facility between April 1, 2005 and April 30, 2022. The study protocol was approved by the ethics committee of the hospital (No. 22-284).

### Participants and data collection

All patients with ETB were eligible, and exclusion criteria were not established. Data regarding age, sex, underlying diseases, presence of fever, digestive symptoms or sepsis at diagnosis, month of diagnosis, source of bacteremia, use of empiric and targeted antibiotics, duration of treatment, and outcomes were extracted from electronic medical records. In accordance with the Surviving Sepsis Campaign [7], sepsis was defined as quick sequential organ failure assessment (SOFA) score ≥ 2. In addition, we also collected data on food and animal exposure from within a week before the diagnosis of ETB. After collecting patient information, we calculated the Charlson’s comorbidity index (CCI) [8]. The occurrence of ETB in the same patient was counted as one case each if the interval was more than 1 month. We described and compared the clinical characteristics, and 30- and 90-day overall mortality rates of patients with ETB.

### Microbiological examination

Blood samples were processed using BacT/ALERT^®^ (bioMérieux, France) until July 31, 2015, and using BACTEC^®^ (Becton Dickinson Diagnostic Instrument Systems, USA) from August 1, 2015 onwards. Identification and antibiotic susceptibility tests were performed using the Walkaway^®^ automated system (Beckman Coulter, Brea, CA, USA). The susceptibility testing panel was changed twice on January 4, 2014 and May 1, 2018. Antibiotic susceptibility was assessed based on the category of Enterobacteriaceae in the M-100, S-26 version of the Clinical and Laboratory Standards Institute (CLSI). For all cultured bacteria, CLSI recommendations and criteria were used to define susceptibility to antimicrobial agents. Polymicrobial bacteremia was defined as a condition wherein more than two species of bacteria, including *E. tarda*, were concurrently identified in blood cultures.

### Statistical analysis

We conducted Fisher exact tests on dichotomous variables and Mann–Whitney *U* tests on continuous variables, and considered p < 0.05 to be statistically significant. All statistical analyses were performed with EZR (Saitama Medical Center, Jichi Medical University, Saitama, Japan), which is a graphical user interface for R (The R Foundation for Statistical Computing, Vienna, Austria). More precisely, it is a modified version of R commander designed to add statistical functions frequently used in biostatistics.

## Results

During the study period, positive blood cultures were obtained in 9368 sets; of which *E. tarda* was detected in 38 cases (0.4%) among the 37 patients.

The patients’ characteristics are presented in Table 1, and detailed in Table 2. There were 22 (57.9%) men and 16 (42.1%) women with a median age of 77.5 years (IQR 74–83, range 25–98). Fever (≥ 37.5 °C) and appetite loss were the common symptoms at diagnosis (68.4% [26/38] and 44.7% [17/38], respectively). One patient (2.6%, 1/38) had diarrhea, and six (15.8%, 6/38) felt nausea or vomited when bacteremia occurred. Sepsis occurred in nine patients (23.7%, 9/38). Data on environmental exposure was available in nine cases for animal exposure and in seven cases for food exposure, where one in nine had a goldfish and a turtle and one in seven had consumed raw fish (Sushi). There was no obvious difference in the incidence of ETB among months − 22 (57.9%) cases during Japanese summer and autumn months (June to November) and 16 (42.1%) cases during winter and spring months (December to May) (Additional file 1: Fig. S1). In only one patient, ETB occurred twice and the source of bacteremia was cholangitis.

**Table 1:** Characteristics of patients with *Edwardsiella tarda* bacteremia

Patient	Total *n* = 38 (%)	Survivors at 30 d, *n* = 32 (%)	Death within 30 d, *n* = 3 (%)	*p* value	Survivors at 90 d, *n* = 23 (%)	Death within 90 d, *n* = 8 (%)	*p* value
Age (median) [range]	77.5 (25–98)	76.5 (25–92)	83 (79–85)	0.111	75 (25–87)	79 (70–90)	0.13
Sex, no. of patients							
Male	22 (57.9)	19 (59.4)	2 (66.7)	1.00	13 (56.5)	6 (75.0)	0.433
Female	16 (42.1)	13 (40.6)	1 (33.3)		10 (43.5)	2 (25.0)	
Symptoms, no. of patients							
Fever (≥ 37.5 °C)	26 (68.4)	22 (68.8)	2 (66.7)	1.00	14 (60.9)	7 (87.5)	0.222
Appetite loss	17 (44.7)	12 (37.5)	3 (100)	0.069	7 (30.4)	6 (75.0)	0.043
Nausea/vomiting	6 (15.8)	6 (18.6)	0 (0)	1.00	4 (17.4)	0 (0)	0.55
Proceeded diarrhea	1 (2.6)	1 (3.1)	0 (0)	1.00	0 (0)	1 (12.5)	0.258
Sepsis	9 (23.7)	8 (25.0)	1 (33.3)	1.00	2 (8.7)	5 (62.5)	0.006
Underlying disease*							
Solid tumor							
Hepatobiliary or pancreatic cancer	12 (31.6)	9 (28.1)	2 (66.7)	0.227	5 (21.7)	5 (62.5)	0.074
Gastrointestinal cancer	6 (15.8)	5 (15.6)	1 (33.3)	0.442	3 (13.0)	2 (25.0)	0.583
Lung cancer	4 (10.5)	4 (12.5)	0 (0)	1.00	3 (13.0)	1 (12.5)	1.00
Prostate cancer	2 (5.3)	2 (6.3)	0 (0)	1.00	2 (8.7)	0 (0)	1.00
Laryngeal cancer	3 (7.9)	3 (9.4)	0 (0)	1.00	3 (13.0)	0 (0)	0.55
Hematologic malignancy	1 (2.6)	1 (3.1)	0 (0)	1.00	1 (4.3)	0 (0)	1.00
Receipt of anticancer chemotherapy	11 (28.9)	10 (31.2)	1 (33.3)	1.00	6 (26.1)	2 (25.0)	0.676
Receiving palliative care	6 (15.8)	3 (9.4)	2 (66.7)	0.047	0 (0)	5 (62.5)	0.0003
Metastatic tumor	6 (15.8)	5 (15.6)	1 (33.3)	0.442	2 (8.7)	4 (50.0)	0.026
Bile duct stent or drainage tube placement	8 (21.1)	5 (15.6)	2 (66.7)	0.095	4 (17.4)	3 (37.5)	0.335
Diabetes mellitus	10 (26.3)	10 (31.3)	0 (0)	0.542	8 (34.8)	2 (25.0)	1.00
Hypertension	18 (47.4)	16 (50.0)	1 (33.3)	1.00	11 (47.8)	4 (50.0)	1.00
Cardiovascular disease	4 (10.5)	3 (9.4)	0 (0)	1.00	3 (13.0)	0 (0)	0.55
Gallstone	17 (44.7)	14 (43.8)	0 (0)	0.259	12 (52.2)	1 (12.5)	0.095
Chronic liver disease**	4 (10.5)	4 (12.5)	0 (0)	1.00	3 (13.0)	0 (0)	0.55
Chronic lung disease***	7 (18.4)	6 (18.6)	0 (0)	1.00	6 (26.1)	0 (0)	0.298
Chronic kidney disease****	3 (7.9)	2 (6.3)	0 (0)	1.00	2 (8.7)	0 (0)	1.00
Dementia	3 (7.9)	1 (3.1)	1 (33.3)	0.166	0 (0)	2 (25.0)	0.060
Stroke	7 (18.4)	6 (18.6)	0 (0)	1.00	2 (8.7)	2 (25.0)	0.268
Receipt of any immunosuppressant*****	2 (5.3)	2 (6.3)	0 (0)	1.00	2 (8.7)	0 (0)	1.00
Connective tissue disease	1 (2.6)	1 (3.1)	0 (0)	1.00	1 (4.3)	0 (0)	1.00
Charlson comorbidity index (median) (range)	5 (0–11)	5 (0–11)	7 (5–10)	0.189	5 (1–11)	8.5 (5–10)	0.002
Types of bacteremia							
Monomicrobial bacteremia	22 (57.9)	19 (59.4)	1 (33.3)	0.565	14 (60.9)	3 (37.5)	0.412
Polymicrobial bacteremia	16 (42.1)	13 (40.6)	2 (66.7)	0.565	9 (39.1)	5 (62.5)	0.412
Source of bacteremia							
Hepatobiliary							
Cholangitis	21 (55.7)	17 (53.1)	2 (66.7)	1.00	11 (47.8)	5 (62.5)	0.685
Cholecystitis	7 (18.4)	5 (15.6)	0 (0)	1.00	4 (17.4)	0 (0)	0.55
Liver abscess	4 (10.5)	4 (12.5)	0 (0)	1.00	4 (17.4)	0 (0)	0.55
Gynecologic	2 (5.2)	2 (5.7)	0	1.00	1 (4.3)	0 (0)	1.00
Intraabdominal abscess	3 (7.9)	2 (5.7)	1 (33.3)	0.242	0 (0)	2 (25.0)	0.013
Appendicitis	1 (2.6)	1 (3.1)	0 (0)	1.00	0 (0)	0 (0)	1.00
Spontaneous bacterial peritonitis	1 (2.6)	1 (3.1)	0 (0)	1.00	0 (0)	0 (0)	0.258
Febrile neutropenia	1 (2.6)	1 (3.1)	0 (0)	1.00	1 (4.3)	0 (0)	0.258
Unknown	1 (2.6)	1 (3.1)	0 (0)	1.00	0 (0)	1 (12.5)	0.258

*With duplicates

**Defined as cirrhosis, hepatitis B virus, or hepatitis C virus infection

***Including chronic obstructive pulmonary disease, intestinal pneumonia

****Defined as a serum creatinine level ≥ 2.0 mg/dL

*****Taking or administrating immunosuppressants or immunomodulators, such as prednisolone

**Table 2:** Detailed characteristics of 38 patients with *Edwardsiella tarda* bacteremia

	Age / Sex	Source of bacteremia / Intervention (if any)	Concurrent organisms from blood culture	Underlying disease	Metastatic cancer	CCI	Animal / Food exposure	Sepsis	Antibiotics	Treatment duration (d) Total (i.v. + p.o.)	30-day survival after bacteremia	90-day survival after bacteremia
1	79 / F	Cholangitis / ERCP (stent)	*K. oxytoca*, *E. cloacae*	Gallbladder cancer (stenting, palliative care), HT	Colon, Pancreas	9	No / No	Yes	C/S → C/S	14 (14 + 0)	Yes	No (at 39 d)
2	63 / F	Febrile neutropenia	None	Acute myeloid leukemia (chemotherapy)		4	No / No	No	VCM, IPM/CS, CAZ → CAZ	14 (14 + 0)	Yes	Yes
3	82 / M	Cholecystitis / PTGBD	None	Gallstone		4	Unknown / Unknown	No	CMZ → Unknown	Unknown	Unknown	Unknown
4	69 / F	Cholangitis / ERCP (ERBD)	*E. coli*, *K. pneumoniae*	Endometrial cancer (chemotherapy, post surgery), Gallstone	None	4	Unknown / Raw fish (Sushi)	No	CMZ → CMZ → CEX (p.o.)	16 (9 + 7)	Yes	Yes
5	42 / F	Ovarian abscess	None	None		0	Unknown / Unkown	Yes	FMOX, CLDM → CMZ → LVFX (p.o.)	17 (12 + 5)	Yes	Unknown
6	67 / M	Cholangitis	*E. casseliflavus*	Laryngeal cancer (chemotherapy), DM, HT	None	5	No / Unknown	No	CMZ → A/S	16 (16 + 0)	Yes	Yes
7	82 / M	Bacterial translocation	None	Rectal cancer (before surgery), DM, Hypertension	Liver	11	Unknown / Unknown	No	CTRX → CMZ	21 (21 + 0)	Yes	Yes
8	76 / F	Liver abscess	None	Lung cancer (chemotherapy/surgery), Laryngeal cancer (surgery), Gallstone, Chronic lung disease	None	4	Unknown / Unknown	No	CMZ → CMZ → LVFX (p.o.)	28 (14 + 14)	Yes	Yes
9	83 / M	Cholangitis / ERCP (stent)	*K. pneumoniae*, *C. perfringens*	Gastric cancer (endoscopic surgery), Stroke, HT	None	5	Unknown / Unknown	No	A/S → CMZ	14 (14 + 0)	Yes	Unknown
10	77 / F	Liver abscess	*Prevotella* sp.	Lung cancer (chemotherapy/surgery), Laryngeal cancer (surgery), Gallstone, Chronic lung disease	None	4	Unknown / Unknown	No	CMZ → CMZ → LVFX (p.o.)	28 (14 + 14)	Yes	Yes
11	80 / M	Cholangitis	*E. coli*	Cholangiocarcinoma (stenting, radiation therapy), Chronic lung disease, ITP (PSL 5 mg)	None	7	Unknown / Unknown	Yes	C/S → C/S → LVFX (p.o.)	23 (6 + 17)	Yes	Yes
12	75 / M	Cholecystitis / Surgery	None	Colorectal cancer (pre-existing condition), Gallstone	None	3	Unknown / Unknown	No	A/S → A/S → A/C (p.o.)	14 (10 + 4)	Yes	Yes
13	75 / F	Cholangitis	None	Gallstone, HT, DM		4	Unknown / No	No	CMZ → CMZ → LVFX (p.o.)	11 (4 + 7)	Yes	Yes
14	85 / F	Cholangitis / ERCP (stent)	*K. pneumoniae*	Pancreatic cancer (stenting, palliative care), HT	Liver	10	No / No	Yes	P/T → P/T	3 (3 + 0)	No (at 3 d)	No
15	77 / M	Cholecystitis / ERCP (stent)	None	Gallstone, Chronic liver disease (HCV)		4	Unknown / Unknown	No	CMZ → CMZ	9 (9 + 0)	Yes	Unknown
16	80 / M	Cholangitis / ERCP (stent)	*E. casseliflavus*, *K. oxytoca*	Pancreatic cancer (chemotherapy, drainage tube placement), DM, HT, Chronic liver disease (HCV)	None	7	Unknown / Unknown	No	IPM/CS → CTX, ABPC	7 (7 + 0)	Yes	Yes
17	80 / M	Cholangitis / ERCP (stent)	*E. aerogenes*, *A. hydrophila*	Pancreatic cancer (chemotherapy, stenting), DM, HT, Chronic liver disease (HCV)	None	7	No / No	No	A/S → A/S	8 (8 + 0)	Yes	Yes
18	68 / M	Cholecystitis / Surgery	None	Gallstone, Chronic liver disease (HBV)		3	Unknown / Unknown	No	C/S → C/S	5 (5 + 0)	Yes	Yes
19	83 / M	Cholangitis ERCP (ERBD)	*E. casseliflavus*, *K. pneumoniae*, *A. hydrophila*	Pancreatic cancer (drainage tube, palliative care), Dementia	None	7	Unknown / Unknown	No	A/S → P/T	12 (12 + 0)	No (at 27 d)	No
20	70 / M	Intra-abdominal abscess / Local drainage	None	Rectal cancer (palliative care)	Liver	9	Unknown / Unknown	Yes	CAZ, MNZ → CAZ, MNZ	14 (7 + 7)	Yes	No (at 73 d)
21	75 / M	Cholangitis / PTCD	None	Pancreatic cancer (before chemotherapy), HT, DM, Stroke	None	7	Unknown / Unknown	Yes	IPM/CS → IPM/CS	12 (12 + 0)	Yes	No (at 54 d)
22	74 / M	Spontaneous bacterial peritonitis	None	Gastric cancer (diagnosed at admission), DM	Liver	9	Unknown / Unknown	No	CTRX → CTRX → LVFX (p.o.)	11 (4 + 7)	Yes	Yes
23	68 / M	Cholangitis / ERCP (stent)	*E. coli*	Cardiovascular disease, Stroke, HT, Gallstone		3	Unknown / Unknown	No	CMZ → CMZ → A/C (p.o.)	14 (7 + 7)	Yes	Yes
24	89 / F	Cholangitis / ERCP (stent)	*E. coli*, *K. oxytoca*	Gallstone, Cholangitis (stenting), Cardiovascular disease, Chronic kidney disease	None	5	Unknown / Unknown	Yes	MEPM → CMZ → TMP/SMX (p.o.)	13 (9 + 4)	Unknown	Unknown
25	74 / M	Cholangitis, Cholecystitis / Surgery	None	Prostate cancer (surgery), Gallstone, Cholangitis, Cardiovascular disease, HT, Chronic lung disease	None	4	Unknown / Unknown	Yes	A/S → CMZ → CEX (p.o.)	15 (12 + 3)	Yes	Yes
26	75 / M	Cholangitis	None	Cholangiocarcinoma (drainage tube), DM, HT	None	6	No / Unknown	No	CMZ → CMZ → A/C (p.o.)	14 (6 + 8)	Yes	Yes
27	83 / F	Liver abscess, Cholangitis / ERCP (stent)	None	Gallstone, SLE, HT		5	Unknown / Unknown	No	CMZ → ABPC → AMPC (p.o.)	28 (7 + 21)	Yes	Yes
28	85 / F	Cholecystitis / PTGBD	None	Gallstone, Cholangitis, HT, Stroke		5	Unknown / Unknown	No	MEPM → A/S	10 (10 + 0)	Yes	Yes
29	90 / M	Unknown origin	*K. oxytoca*	Lung cancer (palliative care), Stroke, HT, Dementia, Gallstone	None	8	Unknown / Unknown	Yes	CMZ → CEZ → CFPM	14 (14 + 0)	Yes	No (at 53 d)
30	71 / M	Appendicitis / Surgery	None	Cardiovascular disease, Diabetes, HT, Chronic lung disease, Chronic kidney disease		6	Unknown / Unknown	No	CMZ → CMZ → LVFX (p.o.), MNZ (p.o.)	14 (7 + 7)	Yes	Yes
31	87 / F	Cholangitis	*K. pneumoniae*	HT, Chronic kidney disease, Gallstone		4	Unknown / Unknown	No	CMZ → P/T	25 (25 + 0)	Yes	Yes
32	98 / F	Cholangitis / ERCP (EST)	None	Pancreatic cancer (palliative care), Gallstone, Stroke, HT, Chronic lung disease, Dementia	None	9	Unknown / Unknown	No	A/S → A/S → LVFX (p.o.)	10 (5 + 5)	Unknown	Unknown
33	79 / M	Intra-abdominal abscess, Peritonitis / Surgery	None	Rectal cancer (chemotherapy)	None	5	Unknown / Unknown	No	CMZ → CMZ	15 (15 + 0)	No (at 16 d)	No
34	25 / F	Intrauterine infection / Surgery	None	Sigmoid sinus thrombosis		1	Goldfish, Turtle / No	No	CMZ → CEX (p.o.)	14 (7 + 7)	Yes	Yes
35	78 / M	Cholangitis, Intra-abdominal abscess	*K. pneumoniae*, *E. faecalis*	DM, Pancreatic cancer (chemotherapy),	Liver	10	Unknown / Unknown	No	MEPM	28 (28 + 0)	Yes	No (at 88 d)
36	77 / F	Cholecystitis / PTGBD	None	AIH (PSL 4 mg), Lung cancer (chemotherapy), Cholecystitis	None	5	Unknown / Unknown	No	MEPM	11 (11 + 0)	Yes	Yes
37	83 / M	Liver abscess	*K. pneumoniae*	Prostatic cancer (chemotherapy), Chronic lung disease, Gallstone	None	7	No / Unknown	No	CMZ → A/C (p.o.)	35 (14 + 21)	Yes	Yes
38	92 / F	Cholangitis	None	Gallbladder cancer (surgery), Stroke, HT	None	4	No / Unknown	No	CMZ → CEX (p.o.)	15 (10 + 5)	Yes	Unknown

ERCP, endoscopic retrograde cholangiopancreatography; PTGBD, percutaneous transhepatic gallbladder drainage; ERBD, endoscopic retrograde biliary drainage; PTCD, percutaneous transhepatic cholangiodrainage; EST, endoscopic sphincterotomy

*K. pneumoniae*, *Klebsiella pneumoniae*; *E. cloacae*, *Enterobacter cloacae*; *E. coli*, *Escherichia coli*; *E. casseliflavus*, *Enterococcus casseliflavus*; *C. perfringens*, *Clostridium perfringens*; *K. oxytoca*, *Klebsiella oxytoca*; *E. aerogenes*, *Enterobacter aerogenes*; *A. hydrophila*, *Aeromonas hydrophila*;

HT, hypertension; DM, diabetes mellitus, SLE, systemic lupus erythematosus; PSL, prednisolone; ITP, idiopathic thrombocytopenic purpura; AIH, autoimmune hepatitis;

C/S, cefoperazone/sulbactam; VCM, vancomycin; IPM/CS, imipenem/cilastatin; CAZ, ceftazidime; CMZ, cefmetazole; CEX, cefalexin; FMOX, flomoxef; CLDM, clindamycin; A/S, ampicillin/sulbactam; LVFX, levofloxacin; P/T, piperacillin/tazobactam; CTX, cefotaxime; ABPC, ampicillin; MNZ, metronidazole;

AMPC, amoxicillin; A/C, amoxicillin/clavulanate; MEPM, meropenem; CFPM, cefepime; TMP/SMX, trimethoprim/sulfamethoxazole; p.o., per o.s

The median value of CCI was 5 (IQR 4–7, range 0–11). The most frequent underlying diseases in patients with ETB were solid cancer (65.8%, 25/38), followed by hypertension (47.4%, 18/38) and gallstones (44.7%, 17/38). Of all the solid tumors, half were either hepatobiliary or pancreatic cancers (48.0%, 12/25), followed by gastrointestinal cancer (24.0%, 6/25). Six patients had metastatic tumors. Ten patients with solid tumors and one patient with hematologic malignancy had recently received anticancer chemotherapy. Six patients with solid tumors were under palliative care owing to the difficulties in treating their malignancies. For one patient, colorectal cancer was regarded as a pre-existing condition because the patient had had surgery 20 years ago and cancer had not recurred. Eight patients had received bile duct stent or drainage tube insertion for stenosis arising from malignancies or gallstones in the past. None of the patients had liver cirrhosis.

The sources of bacteremia included cholangitis (55.7%), cholecystitis (18.4%), and liver abscess (10.5%). In addition, other intra-abdominal infections, such as appendicitis, gynecologic infections, febrile neutropenia, and those of unknown origin have been observed in a few cases. Endoscopic or surgical drainage procedures were performed for cholangitis (52.4%), cholecystitis (100%), and liver abscess (25.0%). One patient had diarrhea a few days before developing bacteremia. The cause of bacteremia in this case was cholangitis and not gastroenteritis, because *E. tarda* was detected in bile cultures collected by percutaneous transhepatic biliary drainage. Sixteen patients (42.1%) exhibited polymicrobial bacteremia, with eight of them carrying more than three pathogens. The source of polymicrobial bacteremia was hepatobiliary in fifteen patients, and it was of unknown origin in one patient.

Susceptibility tests were performed on all *E. tarda* samples (Table 3). Almost all specimens were susceptible to all β-lactams, including ampicillin. One strain was resistant to both ampicillin and piperacillin but susceptible to ampicillin-sulbactam, piperacillin-tazobactam, and all cephalosporins. Molecular analysis of drug resistance was not performed. All specimens were susceptible to non-β-lactam agents such as fluoroquinolones, trimethoprim-sulfamethoxazole, and tetracyclines. Since the susceptibility panel utilized in the broth microdilution method did not contain colistin, there were no susceptibility data for it.

**Table 3:** Antibiotic susceptibility of 38 *Edwardsiella tarda* strains from blood culture

Antibiotics	MIC value (μg/mL)									Susceptible (%)	Intermediate (%)	Resistant (%)
	≤ 0.5	1.0	2	4	8	16	16 <	2/38	64 <			
Ampicillin				11*	26**		1			97.4	0	2.6
Piperacillin					37***				1	97.4	0	2.6
Ampicillin-Sulbactam^+^					27*					100	0	0
Piperacillin-Tazobactam^+^						27*				100	0	0
Cefazolin				38***						100	0	0
Cefotaxime		11**			27*					100	0	0
Cefepime^+^			27*							100	0	0
Cefmetazole				11*	27**					100	0	0
Imipenem		38***								100	0	0
Meropenem^+^		27*								100	0	0
Aztreonam				27**	11*					100	0	0
Gentamicin		10**	28*							100	0	0
Levofloxacin	27*	11**								100	0	0
Minocycline		11**	27*							100	0	0
Trimethoprim-Sulfamethoxazole								38***		100	0	0

*MIC* minimum inhibitory concentration

*Lowest MIC including inequality that could be measured on the susceptibility panel after January 4, 2014

**Lowest MIC including inequality that could be measured on the susceptibility panel until January 3, 2014

***Lowest MIC including inequality that could be measured on the susceptibility panel through the study period

^+^Antibiotics that were started to measure MIC after January 4, 2014

The types of antibiotics administered and the duration of treatment are shown in Table 2. All patients were started with intravenous agents. The most commonly administered initial antibiotic was cefmetazole (44.7% [17/38]). Seven patients were initially treated with carbapenems and six of the seven patients were shifted to narrow-spectrum antibiotics, once susceptibility and microbiological results were revealed. Three patients underwent escalation therapy. Patient 19 was switched from ampicillin-sulbactam to piperacillin-tazobactam because *Aeromonas hydrophila* was detected in bile cultures in addition to *E. tarda.* Two patients had complications of other infections during treatment for *E. tarda* infections. In patient 29, cefmetazole was changed to cefepime because of aspiration pneumonia. Patient 31 was switched to piperacillin-tazobactam from cefmetazole due to *Pseudomonas aeruginosa* bacteremia that occurred during treatment of ETB. The median treatment duration for ETB was 14 days (IQR 11–16.5, range 3–35), intravenous administration was for 10.0 days (IQR 7–14, range 3–28), and oral antibiotics administration was for 3.0 days (IQR 0–7, range 0–21) which included fluoroquinolones, trimethoprim-sulfamethoxazole, and β-lactams.

The 30- and 90-day overall mortality rates were 8.6% (3/35) and 25.8% (8/31), respectively (Table 1). While the median CCI for patients alive and dead at 30 days was 5 [range, 0–11] and 7 [range, 5–10] (p = 0.189), it was 5 [range, 1–11] and 8.5 [range, 5–10] (p = 0.002) at 90 days, respectively. Patients 14, 19, and 33 died within 30 days after ETB. ETB-associated death occurred in patient 14 and 33. Patient 19 died due to *Enterobacter aerogenes* bacteremia after being successfully treated for ETB. On the contrary, patients 1, 20, 21, 29, and 35 died between 30 and 90 days, and the deaths occurred after successful treatment for ETB. All these patients had advanced cancer. Except for patient 35, all other patients had received palliative care because their cancers were difficult to treat. Patient 21 died of hemorrhagic shock due to tumor invasion to celiac artery, whereas other patients died due to cancer progression.

## Discussion

We reviewed 38 patients with ETB. The results suggest that ETB is uncommon, gastrointestinal symptoms of bacteremia rarely occur more than previously reported, and the 30-day overall mortality was 8.6%, but the 90-day mortality was increased to 25.8%. The seasonality of ETB could not be confirmed, which is consistent with a previous retrospective study conducted in Japan [5].

Chronic liver diseases such as cirrhosis and iron overload are reported as risk factors for *E. tarda* infection [1], and in cases of ETB, the most common underlying diseases were gallstones (11.5–27.3%) and malignancy (22.1–46.2%) [5, 6]. Our patients commonly had gallstones (44.7%) and malignancies (65.8%), especially hepatobiliary or pancreatic cancer. These might be risk factors for ETB, although we accepted it as experimental because we did not perform a multilogistic analysis.

The incidence of diarrhea was only 2.6% in our patients, although in a previous study, it was reported to be present in 24.7% of patients with ETB [6]. In this study, the most common focus of bacteremia was cholangitis, followed by cholecystitis and liver abscess. Some of our patients had appetite loss, nausea, or vomiting without diarrhea. These symptoms are not specific to enterocolitis and commonly occur in bile duct infections. This suggests that gastroenteritis is an uncommon cause of bacteremia.

The overall 30-day mortality in our patients was 8.6% and 90-day mortality was 25.8%. The low 30-day mortality rate observed in this study might be because of the high proportion of controllable bacteremia, as in cholangitis; the high frequency of interventions, such as endoscopic retrograde cholangiopancreatography or surgical procedures; and the suitability of empiric antibiotics. The 90-day mortality was increased to 25.8%, but all the patients who died between 30 and 90 days had successfully completed treatment for ETB. No ETB-related death occurred. As shown in Table 1, the higher rates of receiving palliative care and metastatic cancer in patients who died within 90 days suggest that unfavorable underlying conditions, rather than ETB itself, might be the reason for the outcome in this study. In a previous literature review [6], liver cirrhosis was considered an independent risk factor for death, and the crude 90-day mortality increased to 61.1% (11/18 cases), especially in patients with infections of the skin and soft tissues. We believe that the difference in mortality between our study and the literature review may be due to the absence of patients with liver cirrhosis and infections of the skin and soft tissues in this study.

β-Lactamases in *E. tarda* might be chromosomally encoded, although it is susceptible to almost all β-lactams other than penicillin and oxacillin. Colistin resistance is present in almost all strains [9, 10]; however, the underlying mechanism is unclear. One strain of *E. tarda* in our study was resistant to both ampicillin and piperacillin, and this susceptibility was recovered by β-lactam/β-lactamase inhibitors. All cephalosporins remained susceptible, suggesting that β-lactamase produced by *E. tarda* might be penicillinase, which has never been reported. Although isolates from humans may not have been reported to have a minimum inhibitory concentration (MIC) ≥ 64 for ampicillin or piperacillin [9–11], the strain and molecular analysis of drug resistance are not available. *E. tarda* isolated from farming fish sometimes has plasmids related to drug resistance [12], which may be reported in humans in the future.

This is the largest single-center investigation that has mentioned the characteristics of ETB, which adds to the knowledge of the clinical features of ETB that are not well understood. However, our study had several limitations. First, this was a descriptive study conducted at a single institution. While the median CCI for patients who died within 90 days of ETB occurrence was higher than that for survivors, high CCI originally implied a poor prognosis [8]. Although ETB might have given a worse prognosis for patients with high CCI, we did not analyze the risk factors for mortality because of the small sample number and some missing data. However, because there might be regional variations in epidemiology, such as incidence of ETB, patients’ characteristics, and mortality, we hope that a nationwide study will be performed regarding the characteristics of ETB in the future. Second, because this was a retrospective review of electronic medical records, exposure to freshwater, seawater, animals, and raw foods was unavailable in most cases. Although patient 34 bred goldfish and turtles, *E. tarda* could not be identified in the water culture in the tank. However, because there are many bacteria in the tank and it is difficult to control the quality of the samples, the culture results are not always reliable.

## Conclusions

Our retrospective review of patients with ETB showed that nearly half of the patients had malignancies or gallstones, and antecedent gastrointestinal symptoms and incidents of bacteremia from gastroenteritis were less common. It also revealed that the 30-day mortality rate for ETB was low, but the 90-day mortality was increased due to the progression of unfavorable underlying diseases. We hope that a larger study will identify risk factors for mortality in patients with ETB.

## Supplementary Information

**Additional file 1: Figure S1.** Number of cases of Edwardsiella tarda bacteremia reported each month.

## Data Availability

All the data relevant to this study will be made available upon reasonable request to the corresponding author.

## Abbreviations

CCI: Charlson comorbidity index
CLSI: Clinical and Laboratory Standards Institute
ETB: *Edwardsiella tarda* Bacteremia
IQR: Interquartile range
MIC: Minimum inhibitory concentration
