# Supplementary material for: Characteristics and prognosis of patients with Edwardsiella tarda bacteremia at a single institution, Japan, 2005–2022
Source: Ann Clin Microbiol Antimicrob. 2022 Dec 7;21:56. doi: 10.1186/s12941-022-00548-w (PMC9730647; doi:10.1186/s12941-022-00548-w)
Supplement: Supplementary file 1 — Additional file 1: Figure S1. Number of cases of Edwardsiella tarda bacteremia reported each month. [file 12941_2022_548_MOESM1_ESM.pptx]

## Slide 1
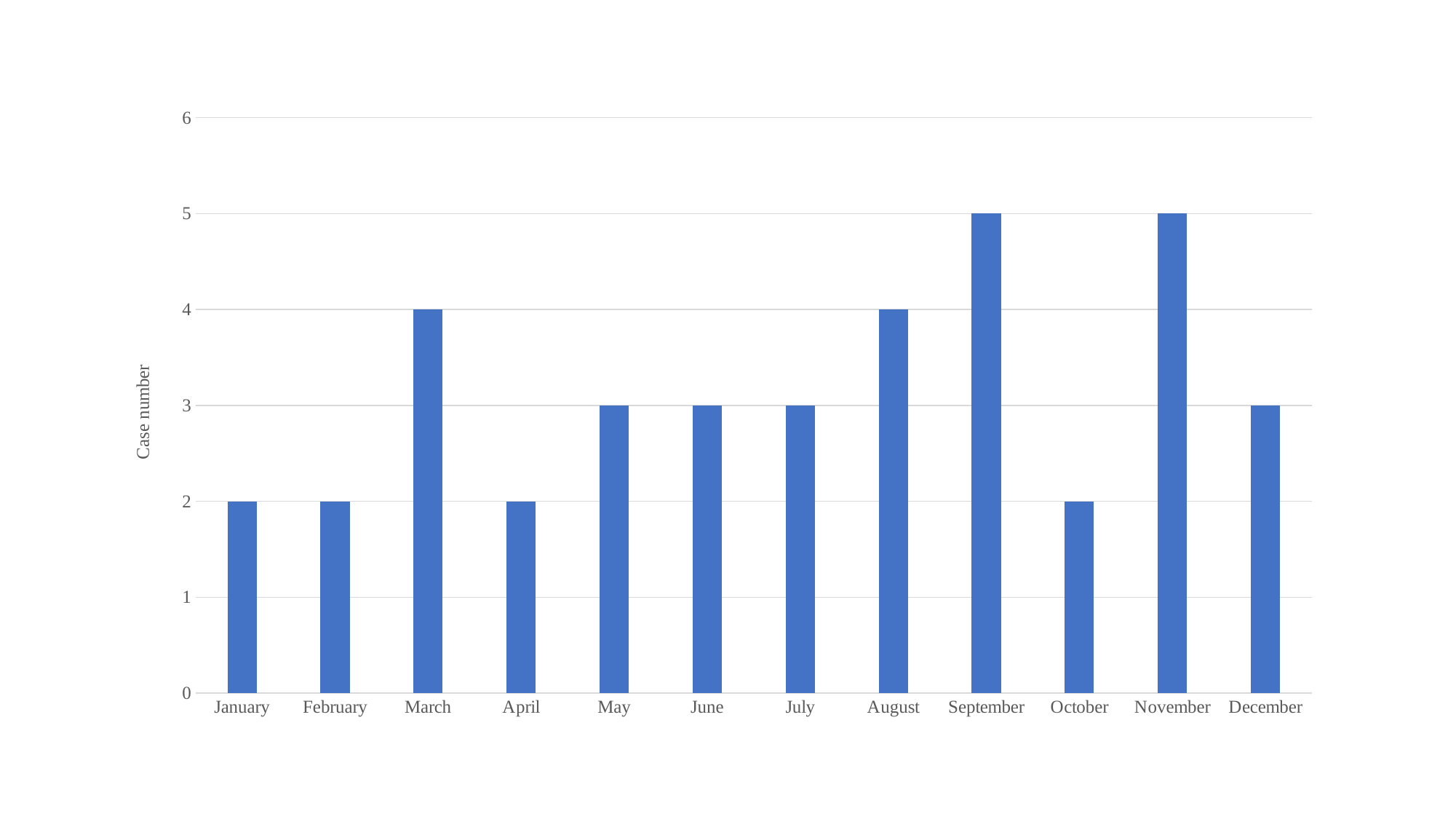

### Chart
| Category | Case number |
|---|---|
| January | 2.0 |
| February | 2.0 |
| March | 4.0 |
| April | 2.0 |
| May | 3.0 |
| June | 3.0 |
| July | 3.0 |
| August | 4.0 |
| September | 5.0 |
| October | 2.0 |
| November | 5.0 |
| December | 3.0 |
